# Author Correction: Pharmacological perturbation of CXCL1 signaling alleviates neuropathogenesis in a model of HEVA71 infection

**DOI:** 10.1038/s41467-022-29362-w

**Published:** 2022-03-24

**Authors:** Saravanan Gunaseelan, Mohammed Zacky Ariffin, Sanjay Khanna, Mong How Ooi, David Perera, Justin Jang Hann Chu, John Jia En Chua

**Affiliations:** 1 Department of Physiology, Yong Loo Lin School of Medicine, National University of Singapore, Singapore, Singapore; 2 Department of Microbiology and Immunology, National University of Singapore, Singapore, 117597 Singapore; 3 LSI Neurobiology Programme, National University of Singapore, Singapore, 117456 Singapore; 4 Department of Paediatrics, Sarawak General Hospital, Kuching, Sarawak, Malaysia; 5 Institute of Health and Community Medicine, Universiti Malaysia Sarawak, Kota Samarahan, Sarawak, Malaysia; 6 Institute of Molecular and Cell Biology, Agency for Science, Technology and Research (A*STAR), Singapore, 138673 Singapore; 7 Infectious Disease Translational Research Programme, National University of Singapore, Singapore, 117597 Singapore; 8 Healthy Longevity Translational Research Program, Yong Loo Lin School of Medicine, National University of Singapore, Singapore, Singapore

**Keywords:** Cell death in the nervous system, Pathogens, Viral infection

Correction to: *Nature Communications*10.1038/s41467-022-28533-z, published online 16 February 2022.

In this article the affiliation ‘Infectious Disease Translational Research Programme, National University of Singapore, 117597, Singapore’ for Justin Jang Hann Chu was missing. The original article has been corrected.

